# ‘We Are Drinking Diseases’: Perception of Water Insecurity and Emotional Distress in Urban Slums in Accra, Ghana

**DOI:** 10.3390/ijerph17030890

**Published:** 2020-01-31

**Authors:** Joseph Kangmennaang, Elijah Bisung, Susan J. Elliott

**Affiliations:** 1 Department of Geography and Earth Sciences, University of North Carolina at Charlotte, Charlotte, North Carolina, NC 28223, USA; 2 School of Kinesiology and Health Studies, SKHS Building 28 Division Street, Queen’s University, Kingston, ON K7L 3N6, Canada; eb120@queensu.ca; 3 Department of Geography and Environmental Management, University of Waterloo, 200 University Avenue West Waterloo, ON N2L 3G1, Canada; susan.elliott@uwaterloo.ca

**Keywords:** emotional distress, vended water, water security, photo-voice, Accra, Ghana

## Abstract

Water security is critical to the health and well-being of people around the world, especially among populations experiencing water stresses and rapid urbanization in low- to middle-income countries (LMICs). Recent research suggests water insecurity is associated with negative mental health outcomes. Despite global improvement in access to safe water across the world, the World Health Organization (WHO) reports that access to safe water in urban areas has not changed significantly or has stagnated in certain countries. In most African cities, entrepreneurial water vendors have stepped up to fill supply gaps in the formal delivery system by selling vended water. As part of a larger research program that aims to assess and analyze public perceptions around vended water, this paper explores the links connecting water insecurity and emotional distress among urban slum dwellers who mostly use vended water in Accra, Ghana. We used a parallel mixed-methods approach. Our quantitative results show that water-insecure households (OR = 2.23, *p* = 0.01) were more likely to report emotional distresses compared to water-secure households. However, households with improved sanitation (OR = 0.28, *p* = 0.01) and those willing to participate for improved water and sanitation (OR = 0.28, *p* = 0.01) were less likely to report emotional distress. Our qualitative results offered support for the quantitative results, as participants not only hold various perceptions regarding the safety and quality of vended water but expressed emotional distresses such as fear of contamination, discomfort, worry over arbitrary change in prices, and anxiety. The implications of the results for policy and practice, specifically to ensuring access to safe water, are discussed.

## 1. Introduction

Globally, an estimated 663 million people remain without access to improved drinking water within a reasonable distance from home and 2.5 billion lack improved sanitation facilities [1]. Even households estimated to have access to improved water sources stand the risk of drinking unsafe water because of the poor microbial quality of some improved water sources [2,3]. This could pose significant risks to health and well-being, since approximately 85 percent of diarrheal diseases among children below age five is attributed to unsafe water and sanitation [4,5]. Aside from microbial infections and other direct health impacts, water insecurity also compromises health and well-being in other ways. Water insecurity has been shown to be associated with distress, frustration, embarrassment and anxiety among individuals, particularly women [6,7,8,9]. Other studies have also revealed that the time and money spent to access water due to insecurity depletes households’ savings and productive time [10].

Water security is complex and recent literature reveals diverse interest in the concept across disciplines. We acknowledge the varied views, academic and policy debates, and the multiple definitions of water security [6,7,11,12,13,14]. However, for this research, we define water security as the ability to access water without stress and to benefit from reliable, affordable, adequate and safe water for healthy lives and well-being (Jepson et al.; 2017). Therefore, water insecurity occurs when any of these variables (stress free access, reliable, affordable, safe) are not met. Further, depending on the social arrangement of an area, water security may entail peoples’ ability to access water through culturally acceptable means [3,9].

In low- to middle-income countries (LMICs), increasing urbanization coupled with weak institutional capacity to expand and maintain water infrastructure affects the reliability and quality of water supply [15]. Another increasing concern is that global interventions on water security have been challenged by the forces of climate change, which affect water availability and quality [16,17]. In most urban areas in sub-Saharan Africa, entrepreneurial water vendors have stepped up to fill supply gaps in the formal/public delivery system by selling water through various mediums [18,19,20]. These mediums include: (1) by a tank or container using trucks, bicycles or hand carts; (2) by selling to households from vendor’s own water, supplied by a public system or from a borehole; and (3) water packaged as bottled or sachet water with varying degrees of filtration or disinfection [18,20,21]. These sources of water may have adverse impacts on the health and well-being of populations including: (1) concerns about microbial contaminants, since sources of vended water are unknown, poorly monitored, and sometimes packaged in unhygienic conditions; (2) high cost of vended water that results in the inability of households to purchase sufficient quantities; and (3) environmental concerns resulting from waste from water bottles and sachets [18,19,20]. For example, close to 14% of urban populations reported sachet water as their main source of drinking water in the 2010 Ghana Census [22]. This poses serious health risks given the poor water handling, hygiene and waste management practices in these countries.

There is extant literature examining the emotional distresses associated with the lack of or scarcity of various resources including water, food and income [22,23,24,25,26,27,28]. Food and water insecurities have been linked to emotional distress, anxiety, and depression in different contexts [29,30]. Emotional distress in this paper refers to feelings of anger, frustration, embarrassment and depression associated with water collection or purchases [25,31,32]. Urban households may experience quarrels, embarrassment or worry when water from these vended sources are inadequate for their household needs, or unwholesome for themselves and their visitors. Further, queuing or competing for safe water negatively affects slum dwellers’ daily life, including their social engagements and social relationships. For instance, water collection from vendors may require women to forgo social gathering to wait for water entrepreneurs or face the wrath of their husband if they fail to secure enough water for household needs. Water collectors are also left at the mercy of water entrepreneurs, who often transfer any supply inadequacies such as breakdown of water infrastructure, inadequate quantities or arbitrary change in prices to households. The lack of control over these daily household needs may negatively impact on the health and well-being of these households. Despite this, there are few studies in LMICs that document the emotional distresses associated with vended water use as well as examine the links between vended water use and water insecurity [25,26,27,28].

This paper contributes to this small but growing body of scholarship on water security and emotional distress in urban informal settlements in LMICs by documenting the experiences of vended water in terms of anxiety, anger and safety using data collected from slum dwellers in Accra, Ghana. We first examine the prevalence of water-related emotional distress among a random sample of 499 household heads. Next, we document the experiences of emotional distresses associated with vended water using photo-voice interviews. Finally, we present a statistical test of the relationship between vended water-related emotional distress and theoretically relevant variables.

As the global community adopts a multi-sectoral approach to water security, as evidenced by the Sustainable Development Goals (SDG), better understanding of the insecurities associated with vended water (as it becomes the only available option for many slum dwellers) will inform and promote equity in the design and supply of water interventions for the most vulnerable populations. Although a number of studies demonstrate important links between water insecurity and psychosocial stress [10,25,31,33], they remain limited as they do not exclusively focus on water insecurity in terms of quality, quantity and access and among vended water users. In this regard, the aim of this study was to: (1) investigate the relationships between water insecurity and emotional distress among vended water users, and (2) document any experiences of emotional distresses associated with vended water among urban slum dwellers in Accra, Ghana.

## 2. Study Context

**Figure 1:**
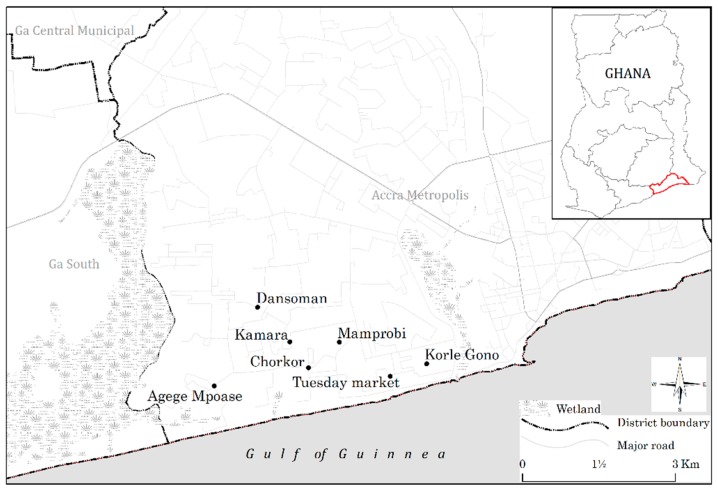
Map of study area.

This study was conducted in urban slums on the shore of the Gulf of Guinea in the Greater Accra Region (GAR) of Ghana. This study covered five urban slums within the Accra metropolitan area in GAR including Agege-Manponse, Korle-Gonnu, Chorkor, Dansoman, and Tuesday Market (see Figure 1). Most slum dwellers in Accra were engaged in economic activities centered on the sea, particularly fishing and petty trading. Even though there has been a general increase in the percentage of households using drinking water from pipped sources across Ghana’s ten (The regions were increased to sixteen in June 2019) administrative regions, pipped water use in GAR dropped from 84.4% to 15.76% between 2000 and 2017 [34]. To fill the supply gaps, the percentage of urban households depending on vended water as their primarily drinking source grew from 9.9% in 2003 to 78.93% in 2017, higher than any of the other regions in Ghana [34]. The GAR also records the fastest population growth in the country, growing at 4.4% annually between 1984 and 2010 due to rural–urban migration [35]. The erratic water delivery in the region may be attributable both to this growth and inadequate investment in water infrastructure [24]. However, the burden of vended water consumption is relatively higher among the urban poor. For instance, slum residents are paying vendors up to eight times the local public utility prices [24,36], and up to twenty times in dryer periods [24]. Fifty percent of households in a sample of Accra’s slum neighborhoods reported using sachets as their primary drinking water source in a 2009–2010 study, and these households tended to be the poorest within the slum communities [24].

## 3. Materials and Methods

This study is cross-sectional in nature and was undertaken between February and April 2018, using a parallel mixed-method design. The qualitative results were used to provide depth to the quantitative results.

### 3.1. Survey

The sampling unit was the household—defined in this context as a person or group of people that live together in same dwelling unit and share domestic resources including food and water [10]. In the first stage, Greater Accra Region (GAR) was purposively selected out of Ghana’s ten administrative regions due to its relatively high levels of urbanization and slum development. According to the 2010 census, the Greater Accra region has approximately 4,010,054 people and is divided into 3666 enumeration areas respectively [22,34]. Within GAR, slum areas within the Accra Metropolitan Area (AMA) were selected based on prior knowledge of the region and relatively high number of slums in this metropolitan area. AMA was divided into clusters guided by the sub-metro divisions and also to ensure that each cluster would provide an adequate number of eligible respondents for inclusion in the survey. The approach both corrects for sampling bias and provides weights to match census percentages of males and females. Households in these clusters were randomly selected for interview.

A household questionnaire was used to assess vended water insecurity, main sources of vended water, access to sanitation, household demographics, household assets, perceived challenges associated with vended water and willingness to participate in community interventions towards water improvement. The survey questionnaire was administered face to face. To ensure the questions were contextual appropriate, a professional translator and one researcher from the University of Ghana translated the questionnaire into Twi, Ga and back to English. Five research assistants (RAs) were recruited and trained to administer the actual survey. These RAs were university graduate students, fluent in Twi or Ga and were familiar with the local context. The RAs also received rigorous training that focused on the research objectives, what each question in the questionnaire sought to measure and general ethical considerations in the data collection process. The questionnaire was pre-tested on 3 March 2017 with 20 people and we obtained satisfactory internal consistency and reliability. The RAs administered the questionnaires with supervision and a debriefing exercise done every evening to evaluate progress and to check for any gaps on completed surveys. Follow-ups were made to correct any gaps that existed. The research was approved by the University of Waterloo Ethics Review Board (22395).

### 3.2. Measures

*Water-related emotional distress:* Similar to [25,31], we measured water-related emotional distresses using six questions that asked respondents the frequency of experiencing outcomes such as quarrels; embarrassment or worry related to drinking or collecting water. Quarrels was measured using two questions that asked respondents to indicate whether in the past 30 days, they or anyone else in the household had a quarrel with neighbors, spouse, household member or relative related to drinking or collecting water. Embarrassment was measured using two questions that asked participants whether they or anyone in the household felt embarrassed to ask for drinking water from a neighbor or felt embarrassed for not being able to provide drinking water for a visitor. Water-related worries were measured using two questions that asked the frequency with which respondents or anyone in their households worried that they could not participate in social activities (e.g., church, mosque, class or school or social gathering) because of drinking water collection duties or felt downhearted or sad that they failed to visit someone or someone failed to visit them because of drinking water collection responsibilities. Based on the responses to these questions, an additive scale was created and categorized into: “0” for those who never experience these events and “1” for people who have experienced at least one psychosocial outcome.

*Water insecurity:* We measured water insecurity using a modified version of the HWIAS developed by [3]. The HWIAS is adapted from the standard Household Food Insecurity Access Scale (HFIAS) [29,37,38] and we have validated our measure of water insecurity among slum dwellers in previous work (see [3,10]).

*Food insecurity:* Food insecurity status was measured using the Household Food Insecurity Access Scale (HFIAS) module, which measures a household’s own perception of their access to food [29,39,40,41]. The Food and Agriculture Organization’s [42] HFIAS scale for measurement of food access indicator guide was used to categorize households into food se-cure, moderately food insecure and severely food insecure. HFIAS has several advantages including the relative ease with which it can be used for data collection compared to other food security measurements such as dietary recalls or anthropometric indicators [29]. Compared to other food security indicators, it captures a higher prevalence rate and correlates well overall with other indicators such as income and education [29,40,41].

*Main source of vended water:* Participants were asked to indicate their main source of drinking water (e.g., bottle, Sachets, bore hole, buying from a private or public vendor, etc.) for members of their household in order of importance from 1 to 3 where 1 is the main source and 3 is the least. We later categorized these sources into three categories: bottle and sachet, buying from a private vendor or buying from a private vendor.

*Perception of vended water:* To measure perception of vended water quality, participants were asked a series of questions to assess how frequently they observed that their drinking water has color; taste; smell; sediments; how frequently they treated drinking water and how frequently they served this water to visitors. These questions capture the WHO guidelines on the aesthetic parameters of water; those detectable by the senses, namely turbidity, color, taste, and odor. These indicators are important in monitoring community water supplies because they influence the adoption of alternative water sources [43,44,45].

*Time spent on water collection and water expenditure:* Time spent collecting water was measured by asking participants the average time in minutes it took them to access their preferred water source. Similarly, water expenditure was captured by asking participants the average amount they spent on water in a day.

*Willingness to participate in collective action:* Based on previous research by [8,10], willingness to participate in collective action to solve water and sanitation issues was created as a Likert scale variable based on the following questions: “how often in the past year have you joined together with others to address a common issue related to water and sanitation in the community”. These common issues included contributing labor for construction of water and sanitation facilities, attending village water committee meetings and contributing cash to a village water and sanitation committee [8]. Based on the responses to these questions, a willingness to participate in collective action scale was created and categorized into willing or unwilling to participate.

*Wealth:* A wealth index was created based on a list of 22 self-reported assets, including number of houses, motorized vehicles and bicycles, animal ownership (e.g., cattle, goats, chicken, etc.), and other household amenities (e.g., fridge, TV, computer, cell phone, etc.). Each asset variable was standardized before principal component analysis was used to calculate a wealth score for each household [46]. The asset score for each household was then weighted by the number of household members, and weighted scores grouped into quintiles.

*Socio-demographics:* We captured household socio-economic status by self-reports of the household head employment status and highest education level. We also included other household demographics such as household size, number of children, number of girls (i.e., females between 6 and 16 years), and number of adult women (females above 16 years). We presumed the number of females in the household might influence experiences of water insecurity and emotional distress as women and girls typically bear the burden of water collection in this context. Further, we included total household members as it is presumed that larger households are associated with competing needs, leading to the likelihood of experiencing emotional distresses.

*Photo-voice interviews:* Photo-voice as a participatory action research method was employed to address our second research objective. Photo-voice is a qualitative method of enquiry built on the principles of social constructivism, community empowerment, education, and documentary photography [8,47]. The concept of photo-voice developed from three main foundations [48,49]. First, it assumes that research and education should start with issues people consider central to their lives and responses to these issues facilitated through active participation and sharing of mutual experiences. Second, photo-voice is underpinned by feminist theory and practice and aims to empower and ensure adequate participation of diverse vulnerable groups including women, children and other minority groups. The method also privileges the lived experiences of these vulnerable groups in the production of knowledge, builds documentary photography and strives to instigate social change by treating participants as active agents in knowledge creation [47,49,50].

## 4. Analysis

### 4.1. Household Survey

All statistical analyses were conducted using the Stata/MP software package, version 15. Based on previous work, household water insecurity was measured using a 7-item modified version of the Household Water Insecurity and Access Scale (HWIAS). We then used Generalized Linear Latent and Mixed Models (Gllamm) with a logit (*log*) link function to build all models: both bivariate and multivariate analysis of emotional distress. Gllamm was employed to correct for bias in the standard errors and parameter estimates due to the hierarchical nature of our survey data, which violates the assumption of independence of respondents in standard logistic regression (see [51,52]). Selection of independent variables for our analysis was influenced by theoretical relevance, data availability, statistical significance, and prior research on emotional distress associated with resource scarcity [10,25,27,33,53]. Model 1 of our analysis controlled for only water-related variables whiles model 2 added socio-demographic variables.

### 4.2. Photo-Voice

The research team first provided each participant with detailed information on the research objectives, basic training in photography skills as well as the ethics of taking photographs. The training was conducted in Ga and Twi (two local languages widely spoken in the study area) and all training manuals and consent forms were also translated into Ga and Twi. A graduate researcher from Korle Bu teaching hospital with prior working knowledge of the community was recruited as the research assistant and acted as translator for this study. After the training exercise, analogue disposable cameras (with 28 exposures each) were given to participants to snap photographs of what they felt best represented their *perception around vended water*. Participants were allowed to take any number of photographs they felt adequately represented their views. All cameras were retrieved after a week and all photographs printed. Overall, participants took between 16–26 photographs, even though not all photographs were related to the objectives of the project but rather photographs of household members and items. Each participant was asked to select five to ten pictures that best represented their perception around vended water. The selected photos were used as a basis for discussion in follow-up individual interviews. In total, ten (*n* = 10) interviews were conducted ranging between 15 and 45 min per photo. During interviews, each participant was generally asked to explain the following regarding the photo: (1) what the photo was and where it was taken; (2) why the photo was important to understanding vended water–water security–health linkages; (3) the relation of the photo to overall health and well-being in the community; and (4) what can be done about the issues or challenges depicted in the photo.

All interviews were audio recorded with permission from participants and transcribed verbatim. The photographs and transcripts were then imported into NVivo 12, a qualitative software package, for analysis. Photographs were initially coded according to the major themes and emerging themes were later added as the coding progressed [8,49]. Some photographs could be classified under more than one category and were thus coded accordingly. The themes, sub-themes and the photographs were reviewed several times to ensure concepts and photographs that related to the same phenomenon and construct were coded under the same category. Data was coded by the lead author, with assistance from other researchers. Emerging codes were organized around four major themes: exposure to contaminants; disease burden on children; psychosocial impacts and infrastructural challenges.

## 5. Results

### 5.1. Quantitative Results

**Figure 2:**
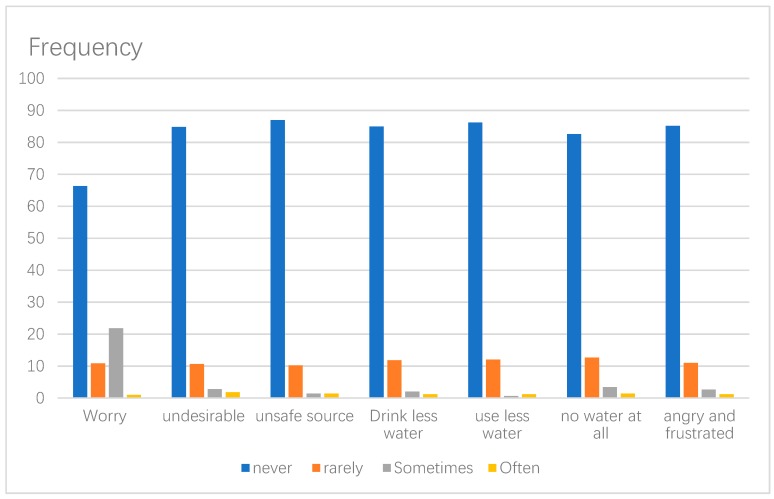
Experiences with water insecurity in the past 30 days in slums in Greater Accra.

A total of 505 households completed the survey out of 550, generating an overall response rate of 91%. Less than 5% of households contained missing data and were pair-wise deleted, generating an analytical sample of 499 households. The sample comprised household heads aged between 18 and 75 years of age (Table 1). The average household size was three, with an average of one child per household. Most household heads were males (51%), with many reporting secondary school as their highest level of educational (43%). Most participants reported Korle-Gonno (33.27%) as their primary place of residence. Approximately 60% of households relied on vended water as their main source of drinking water. However, only 48% perceive vended water to be wholesome. Further, approximately 27% of households reported experiencing emotional stresses related to collecting or buying vended water. Across the neighborhoods, approximately 43% of residents in Chorkor and 33% residents in the other category (Kamara, Mamprobi and Tuesday Market) expressed water-related emotional stresses. More than half of the sample never experienced any of the water insecurity dimensions included in the HWIAS in the past 30 days (Figure 2). Approximately, only one-third of our sample reported not having enough water either rarely, sometimes or often, while one-quarter of households reported drinking water from an unsafe or undesirable source either rarely, sometimes or often (see (Figure 2). A further 21% of all households felt angry and frustrated about the water situation either rarely, sometimes or often. Overall, approximately 42% of households were water insecure. However, most household heads were willing to pay for improved water interventions in their communities. The average time taken to make a round trip of water collection was 16.14 min while the daily average amount spent on water was 2.80 GHC (the equivalent of approximately USD 0.46 at the time of the survey).

### 5.2. Bivariate Association between Emotional Distress and Selected Independent Variables

The results of the bivariate analysis are reported in Table 2. Water-insecure households (OR = 1.90, *p* ≤ 0.01) were more likely to experiences emotional distress compared to water-secure households. Compared to households depending vended water, those depending on other sources (e.g., bore holes, well) (OR = 2.10, *p* ≤ 0.01) were more likely to report emotional distress. Educational level was significantly associated with emotional distress, with women with secondary (OR = 0.27, *p* ≤ 0.01) or higher education (OR = 0.28, *p* ≤ 0.01) being less likely to report experiencing emotional distress. An increase in the number of boys in the household was associated higher likelihood of emotional distress in the household.

### 5.3. Multi-Variate Results

Multivariate results demonstrate that water insecurity, access to sanitation and perception of water quality are associated with emotional distress in this context (see Table 3). Although vended water was related to emotional distress in our bivariate result, it became non-significant in our multivariate results. In model 1 of the multivariate results, households experiencing water insecurity (OR = 1.78, *p* ≤ 0.05) were more likely to report emotional distress compared to water-secure households. Further, households with access to sanitation (OR = 0.28, *p* ≤ 0.01) were less likely to report water-related emotional distresses compared to households without access to sanitary facilities. Other water-related variables associated with emotional distress included: perception of water quality (indicated by smell, color, etc.) and willingness to pay for improve water source. For instance, perception of unwholesomeness of vended water (OR = 2.49, *p* ≤ 0.01) and willingness to pay (OR = 0.24, *p* ≤ 0.01) were associated with higher and lower odds of emotional distresses relatively.

In our final model (model 2), after controlling for *socio-economic and demographic variables*, the association between emotional distress and household water insecurity remained robust. Water-related variables such as water insecurity, perception of water quality, access to sanitation, and willingness to pay remained significant predictors of emotional distress. Socio-demographic factors such as wealth and educational level of the household head were significant predictors of emotional distress. For instance, household heads with secondary education (OR = 0.33, *p* ≤ 0.01) were less likely to be emotionally distressed compared to household heads with no education. However, we found that richer quintiles were more likely to express water-related emotional distress compared to poor households. For instance, the middle quintile (OR = 3.55, *p* ≤ 0.01), rich (OR = 2.24, *p* ≤ 0.05), and richer quintiles (OR = 2.64, *p* ≤ 0.05) were significantly more likely to be emotionally distressed. Demographic variables associated with emotional distress included marital status and region of residence. Separated (OR = 0.43, *p* ≤ 0.05) and married household (OR = 0.54, *p* ≤ 0.05) were less likely to report emotional distress compared to single-headed households.

### 5.4. Qualitative Results

The qualitative results are organized around four key themes: exposure to contaminants, disease burden on children, psychosocial impacts and infrastructural challenges. For ease of reporting, tables are used to illustrate the number of mentions and number of participants who mentioned key themes and sub-themes. The number of photographs under each theme is presented in Table 4. The themes are interspersed with participants’ voices.

### 5.5. Exposure to Contaminants

Some participants expressed concerns with regards to risk of exposure to contaminants as illustrated in the photographs and interviews. The complex links between vended water and environmental risk exposures were demonstrated by many respondents during interviews.

The color of water is like black, dark brown, green…you see like a combination of colors …you can’t really tell…and if you let it sit for some time and you look at the bottom, it doesn’t take long and then it becomes slimy. Father Jesus…you can see that people are suffering…like if you put a cup at the end of the pipe and just drink it, you can see that you’ve taken illness …and people in this house do that thing a lot …they just fetch it and use it for tea…can you just imagine…they are just really killing themselves”

“…if we want to wash or bath, then we have to let it sit and then when it is clear, before you can use it to bath …otherwise you can’t bath with it because your body aches”

### 5.6. Disease Burden on Children

Some participants were also worried about the impacts of unsafe water from pipes on their children. Water from such pipes were regarded as unsafe, inadequate, inappropriate and unacceptable in terms of protecting the health of children:

“It can give us infection…it can trouble your stomach…also, if you cook with it, it cannot do anything good for you…sometimes, the children…when they fetch and drink the water immediately they complain of stomach ache and we cannot even use the water to wash their feeding bottles …we use pure water to wash the feeding bottles”

“…and even in bathing babies…sometimes when we fetch the water and it’s not clean, my mother buys one bag of pure water and then we divide before we use to bath the baby…the nurses told us that if you want to do anything for the baby…you have to get clean and clear water before you do it for the baby”

### 5.7. Psychosocial Impacts

Participants also highlighted the health and social impacts associated with vended water.

“The sachet water too I have a problem with it because sometimes when you finish drinking it and you look inside the sachet rubber, you find that it is slippery…yes… If you pass your fingers through it, it feels like okro …eh hee seriously… after drinking, you realized you have drunk diseases”

“It bothers me because they are several times when I want water to drink water, I have to drink it with the dirt or wait for it to sit (settle), you realize that there are even metal pieces when you fetch it into a white bucket and wait for some days, you’ll see rusting …rust at the bottom of the container …it shows that the water is not good and I worry all the time”

Participants also expressed concerns about arbitrary changes in prices and other costs associated with purifying vended water.

“There are many houses in which there is no pipe and so they go to fetch from other people who sell the pipe water…those who sell the water, if today they sell at 1GHC …after one month or two they will say their bill has increased and you have to pay higher amounts”

“Previously, the water used to be fine, the color was white…but these days they don’t treat the water well (referring to Water vendors and Ghana water company)…because when we fetch the water…you can see that the water is black… brown and others. We have to let the water sit for some time…or you have to go and buy a form (water purifier) before you can drink it”

### 5.8. Infrastructural Challenges

Participants also highlighted that some of the water insecurity challenges were due to dilapidated water infrastructure and poor sanitation. Aside from direct exposure to water contamination due to deteriorating water infrastructure, participants were equally concerned about the lack of maintenance, sensitization and follow up to maintain water infrastructure and educate the public.

“we have to make sure that the people working at where the water is, are trained and the metals …they have to look at the metals(water distribution pipes) …the metals may be very old …they need to change them…I think we get our water from Weija …, Weija is my hometown …if you go there and look at some of the metals…they have to do something”

## 6. Discussion

The aim of this study was to explore perceptions around vended water with respect to emotional distress, safety, quality and affordability among urban slum dwellers in Ghana. This study applied a mixed-methods approach, combining photo-voice interviews with a household survey to better understand perceptions around vended water and its impacts on emotional distress. The combination of both qualitative and quantitative methods enabled us to provide context and in-depth understanding of the links between vended water use and emotional distress among urban slum dwellers who depend on vended water for their household needs. Our qualitative results reveal that participants expressed emotional distresses such fear of contamination, discomfort, worry, and anxiety due to actions and inactions of water entrepreneurs. Participants offered various explanations and justifications for these perceptions including arbitrary changes in prices, poor water infrastructure and maintenance, poor quality of sachet water and delivery gallons, as well as the lack of maintenance and standardization of the activities of water entrepreneurs. The results indicate that both inadequate access and the process of negotiating access led to distress [54]. Gaining entitlement to water via exchanges with water vendors requires women who are tasked with water collection duties in this context to constantly engage in negotiations and arguments about their rights to water access. Our results mirror those of other studies that explore emotional distresses associated with the scarcity of vended water in urban contexts [55,56,57,58,59]. For instance, [56] found, in urban Bolivia, that informal water vendors do not abide by distributive justice (e.g., fair pricing, good water quality), exposing their clients to emotional distress through procedural and interactional injustices. Further, [59] found that water insecurity in urban Lilongwe, Malawi, arises from overdependence on communal water kiosks which are insufficient in number and have low functional rates. Similarly, [60] revealed deep feelings of anxiety and frustration, embarrassment, negative identity, feelings of marginalization, and lack of self-efficacy in Usoma, Kenya. These findings indicate that vended water-related stressors were the consequence of living daily without access to safe water in adequate quantities and the lack of control over supply and pricing.

The multivariate results showed that water-insecure households were more likely to report emotional distresses compared to water-secure households. Our result support previous studies that associate water insecurity in urban slums with various emotional and social impacts [23,24,25,26,61]. Water-insecure households were more likely to report experiencing quarrels, embarrassment or worry related to drinking or collecting vended water compared to water-secure households, and the relationship remained even after controlling for theoretically relevant covariates. The opportunity costs, uncertainties and lost social interactions due to water collection duties may be associated with poor psychological well-being, particularly for households who have to constantly negotiate with water vendors about their right to access safe water. For instance, water collection from vendors may require women to forgo social gathering to wait for water entrepreneurs, or chase after vendors in order to obtain adequate quantities of water [31]. Water collectors are also left at the mercy of water entrepreneurs, who often transfer any supply inadequacies such breakdown of their infrastructure, inadequate quantities or arbitrary change in prices to households. The failure to secure enough quantities of safe water furthers affect the mental and emotional well-being of women as they constantly worry about the quality and safety of alternative water sources used to cook for their families.

Similar to other studies, we found that civic participation and willingness to participate in civic action may be associated with water-related emotional distress. The idea is that people who participate in civic activities are likely to be well informed about Water, Sanitation and Health (WASH) challenges and efforts being made at the community level to improve the WASH situation, leading to proper appraisal of the situation and hope that things will improve. Further, participation in civic activities may be associated with pragmatic acceptance of the water and sanitation situation due to proper appraisal of the WASH situation vis-à-vis available resources and support [62,63,64]. This suggests that sociopolitical processes of engagement, governance, or water citizenship shape emotional distress that results from lack of material resources such as access to water and sanitation. Our findings extend those of other studies that have found that civic engagement maybe associated with emotional distress [8,32,61,65]. Thus, understanding the emotional distresses associated with inadequate access to resources may enrich current policy and programmatic discussions around willingness to participate in collective action as a viable means to upraise and improve water and sanitation in urban slums.

There are inherent limitations associated with this study which are worth acknowledging and should be taken into consideration when interpreting the findings of this study. First, with regards to our photo-voice interviews, participants taking the photos may be biased, as they decided which photos to take or avoid taking [49]. Further, since the participants could choose only five to ten of the photographs to discuss, we may have overlooked some useful information in the other photos. With regards to the household survey, the cross-sectional nature of the study design did not allow us to take into consideration changes and seasonality associated with access to water in the Ghanaian context. Seasonal variations in access to water could also influence household experiences of water insecurity, dependence on vended water as well as time and amount spent on water collection [3,10,31]. Second, our household water insecurity and emotional distress questions were related to participants experiences within the past 30 days and two weeks respectively, which could potentially be affected by recall bias. Third, we acknowledge that men are mostly not active water collectors in the study context and their responses could be biased or inaccurate [10]. However, to correct any potential biases, respondents were asked whether they were in a position to give accurate answers before the interviews started. Research assistants were also encouraged to validate responses with other household members if necessary. Although evidence suggests that women bear a greater responsibility for water collection than men in Accra, and many parts of Ghana, buying water from vendors may affect the entire household budget including financial burden on men. In future studies, it will be important to consider intra-household negotiations and bargaining to purchase vended water. Such negotiations between spouses may have profound implications for the psychosocial well-being of women. For instance, although not reported in our study, studies have found instances of domestic abuse against women when they are unable to provide water to meet household needs [33]. Such problems within relationships point to likely gender dynamics in psychosocial stressors, especially in places where women and girls are culturally obliged to collect water for household use [33]. We believe the social and environmental contexts counts considerably when it comes to stress manifestation. Future research will benefit from understanding the gender dynamics in water-related civic engagements as previous research indicates women appear to be more driven to engage in community action by water quality and men by access [10,61]. Fourth, this study focused on primary household water uses and did not include the full range of water uses within the household.

## 7. Conclusions

Notwithstanding these limitations, this study provides strong evidence for the emotional distresses associated with vended water use and contributes to the literature on water-related emotional distresses, especially in urban settlements in low- to middle-income countries. The findings also have implications for Ghana and other sub-Saharan African countries as they begin to implement the SDGs, highlighting the need to set up and enforce vended water standards and regulation of the activities of water entrepreneurs. Finally, this study emphasizes the importance of targeting both the material conditions of lack of access to safe water within urban slums as well as the psychosocial and mental response of vulnerable populations facing inadequate access to safe water and advocates for a holistic approach to water insecurity beyond physical health outcomes to include its social and emotional impacts.

## Figures and Tables

**Table 1:** Descriptive statistics.

Independent Variables	Greater Accra
	Frequency (%)
Emotional distress	
None	353 (72.63)
One or more	133 (27.37)
Water security	
Secure	290 (58.12)
Insecure	209 (41.88)
Perception of water quality	
Wholesome	261 (52.30)
Unwholesome	238 (47.70)
Main source of drinking water	
Sachet/bottle	425 (85.14)
Others	74 (14.83)
Distance to collect water (mean)	16.14 (0–100)
Average amount spent daily on water (Ghana Cedis)	2.80 (0.2–50)
Perception of water quality	
Unwholesome	261 (52.30)
Wholesome	238 (47.70)
Adequate water for household needs	
Inadequate	146 (29.26)
Adequate	353 (70.74)
Sanitation facility	
Unimproved	341 (68.34)
Improved	158 (31.66)
Willingness to pay for community water interventions	
Unlikely	352 (70.54)
Likely	147 (29.46)
Household food security	
Secure	185 (37.07)
Insecure	314 (62.93)
Household wealth level	
Poor	107 (21.44)
Middle	129 (25.85)
Rich	100 (20.04)
Richer	70 (14.03)
Richest	93 (18.64)
Education level	
None	71 (14.23)
Primary	106 (21.24)
Secondary	215 (43.09)
Tertiary	107 (21.44)
Sexual	
Male	255 (51.10)
Female	244 (48.90)
Marital status	
Single	188 (37.68)
Separated	61 (12.22)
Married	250 (50.10)
Household size (mean)	2[1–18]
Number of girls under 18 in the household (mean)	1[0–10]
Number of boys in the household (mean)	1[0–12]
Number of adult women in the household (mean)	2[1–17]
Number of adult men in the household (mean)	2[1–17]
Neighborhoods of residence	
Agege-Manponse	60 (12.02)
Chorkor	157 (31.46)
Dansoman	58 (11.62)
Korle Gonno	166 (88.38)
Other	58 (11.62)
Observations	499

**Table 2:** Bivariate associations between emotional distress and selected independent variables.

Independent Variables	OR (95% CI)
Water insecurity (ref: secure)	
Insecure	1.90 (1.22–2.95) ***
Main source of drinking water (ref: sachet or bottle)	
Others	2.10 (1.24–3.55 ***
Distance to collect water	1.01 (1.000–1.012) *
Amount spent	0.92 (0.82–1.03)
Quantity of water (ref: inadequate)	
Adequate	1.62 (1.01–2.61) **
Perception of water quality (ref: wholesome)	
Unwholesome	1.71 (1.06–2.74) **
Access to sanitation (ref: unimproved)	
Improved	0.26 (0.15–0.47) ***
Willingness to pay for community water interventions (ref: unlikely)	
Likely	0.27 (0.16–0.47) ***
Household food security status (ref: secure)	
Insecure	1.53 (0.98–2.39) *
Household wealth level (ref: poor)	
Middle	1.69 (0.94–3.03) *
Rich	1.32 (0.68–2.55)
Richer	1.28 (0.63–2.60)
Richest	0.71 (0.35–1.43)
Educational level (ref: none)	
Primary	0.55 (0.28–1.08) *
Secondary	0.27 (0.15–0.51) ***
Tertiary	0.28 (0.14–0.59) ***
Sex (ref: male)	
Female	0.92 (0.61–1.39)
Marital status (ref: single)	
Separated	0.72 (0.36–1.43)
Married	0.72 (0.45–1.16)
Household type (ref: Nuclear)	
Extended family	0.93 (0.57–1.51)
Number of Household members	1.07 (0.96–1.19)
Number of girls under 18 in the household	1.15 (0.99–1.34) *
Number of boys under 18 in the household	1.22 (1.05–1.42) ***
Observations	499

Notes: OR = odds ratio; Ref: = Reference Categories; * *p* ≤ 0.10, ** *p* ≤ 0.05, and *** *p* ≤ 0.01; CI = confidence intervals.

**Table 3:** Multivariate associations between emotional distress and selected independent variables.

Independent Variables	Model 1 OR(SE)	Model 2 OR(SE)
	Emotional Distress	Emotional Distress
Water insecurity (ref: secure)		
Insecure	1.78 (1.04–3.04) **	2.24 (1.25–4.01) ***
Main source of drinking water (ref: sachet or bottle)		
Other	1.60 (0.91–2.82)	1.29 (0.69–2.41)
Distance to collect water	1.01 (1.00–1.01) *	1.01 (0.99–1.01) *
Amount spent	0.94 (0.84–1.06)	0.96 (0.87–1.07)
Quantity of water (ref: inadequate)		
Adequate	0.89 (0.51–1.58)	1.13 (0.61–2.12)
Perception of water quality (ref: wholesome)		
Unwholesome	2.49 (1.48–4.19) ***	2.23 (1.27–3.93) ***
Access to sanitation (ref: unimproved)		
Improved	0.28 (0.16–0.50) ***	0.29 (0.15–0.54) ***
Willingness to pay for community water interventions (ref: unlikely)		
Likely	0.28 (0.16–0.51) ***	0.28 (0.15–0.53) ***
Household food security status (ref: secure)		
Insecure		1.43 (0.85–2.43)
Household wealth level (ref: poor)		
Middle		3.55 (1.77–7.12) ***
Rich		2.24 (1.04–4.85) **
Richer		2.64 (1.15–6.08) **
Richest		2.05 (0.87–4.81) *
Educational level (ref: none)		
Primary		0.64 (0.31–1.35)
Secondary		0.33 (0.16–0.67) ***
Tertiary		0.51 (0.22–1.17)
Sex (ref: male)		
Female		1.02 (0.630–1.661)
Marital status (ref: single)		
Separated		0.44 (0.19–0.97) **
Married		0.50 (0.28–0.87) **
Household type (ref: Nuclear)		
Extended family		0.92 (0.50–1.67)
Household number		0.94 (0.76–1.17)
Number of boys under 18		1.24 (0.95–1.61)
Number of boys under 18		0.98 (0.76–1.28)
**Random effects**		
Neighborhood	1.00 (0.73–1.36)	1.00 (0.72–1.38)
Constant	0.32 (0.16–0.66) ***	0.31 (0.08–1.15) *
**Observations**		499

Notes: OR =odds ratio; Ref: = Reference Categories; * *p* ≤ 0.10, ** *p* ≤ 0.05, and *** *p* ≤ 0.01; CI = confidence intervals.

**Table 4:** Thematic summary of photos.

Impacts	# of photographs (*n* = 30)
Exposure to contaminants	10
Disease burden on children	6
Psychosocial health	10
Infrastructural challenges	4
